# Commentary: Flow cytometry quantification of tumor-infiltrating lymphocytes to predict the survival of patients with diffuse large B-cell lymphoma

**DOI:** 10.3389/fimmu.2024.1377221

**Published:** 2024-04-18

**Authors:** Zhongling Sun, Ran Tan, Huanling Wu, Xiaosheng Fang

**Affiliations:** ^1^ Department of Neurology, Zhaoyuan People’s Hospital, Zhaoyuan, China; ^2^ Department of Hematology, Shandong Provincial Hospital Affiliated to Shandong First Medical University, Jinan, Shandong, China; ^3^ Department of Laboratory Medicine, Shandong Provincial Hospital Affiliated to Shandong First Medical University, Jinan, Shandong, China

**Keywords:** flow cytometry, TIL-B, tumor-infiltrating lymphocytes, TME, TIL, DLBCL, prognosis, clinical decision support tool

## Introduction

**Figure 1:**
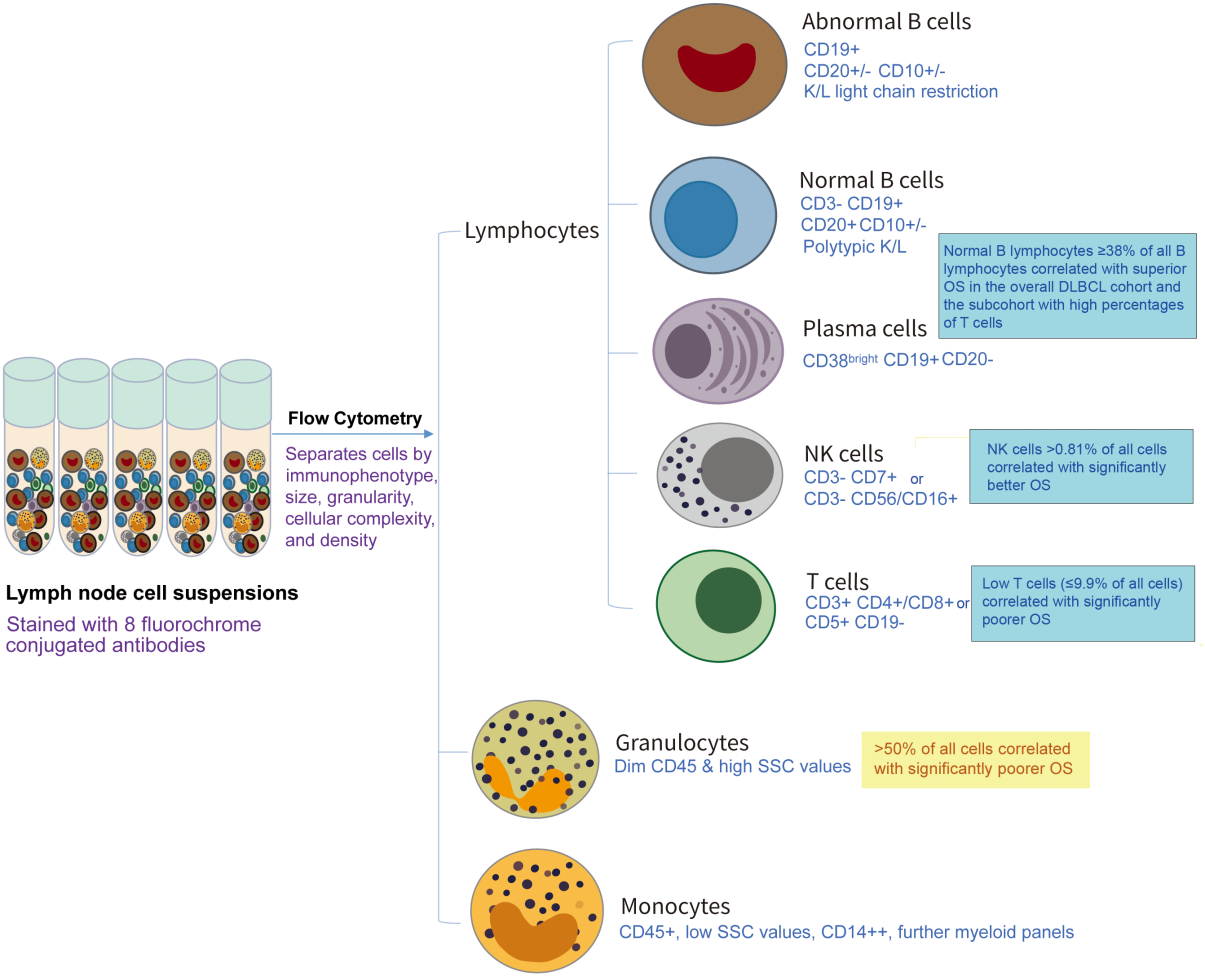
Schematic illustration of the prognostic values of tumor-infiltrating immune cells by flow cytometry analysis in a recent study (Ref. 6) by Yu et al. in Front Immunol 2024, Volume 15, doi: 10.3389/fimmu.2024.1335689. In that study, cell populations were identified and quantified by various antibody panels and different gating strategies by clinical workups according to the Duke Clinical Flow Cytometry Laboratory Standard of Operating Procedures in 2011-2022, and this figure only lists key markers used to identify different immune cell types as summarized in Reference 6.

Immune cells responsible for anti-tumor surveillance are often inhibited or dysfunctional in cancer, yet they remain to be critical for the clinical outcome of chemotherapies and immunotherapies (1–3). In clinical practice, immune cells are quantified by flow cytometry for the diagnosis of hematologic malignancies, including leukemia, myeloma, and lymphoma (4, 5). In the article collection of “Tumor Microenvironment and Hematological Malignancies: New Evidences and New Questions” in *Frontier in Immunology*, Yu and colleagues at Duke University Medical Center collected flow cytometry data for fresh diagnostic biopsies from 102 patients with *de novo* diffuse large B-cell lymphoma (DLBCL), the most common lymphoma, and demonstrated the favorable prognostic effects of tumor-infiltrating lymphocytes (TILs) in DLBCL (**Figure 1**). To predict clinical outcome, independent prognostic factors were used to build a LASSO-Cox model and a graphic tool (6), demonstrating the proof-of-concept that clinical flow cytometry data can be used for risk prediction in DLBCL, as well as the prognostic significance of immune cell functions in the context of DLBCL disease with various immune escape mechanisms and treatments (7, 8).

## Clinical flow cytometry data to predict prognosis and facilitate treatment decision

The prognostic role of the tumor microenvironment (TME) in DLBCL has been demonstrated by independent studies using different approaches at the DNA (9), RNA (10, 11), and protein (12) levels. Notably, methods used in these previous studies are generally for researches only; it is important to develop a clinical decision support tool for tailoring personalized regimens and selection of immunotherapy. The flow cytometry study by Yu et al. (6) showed that integrating TME data into risk prediction is feasible in clinical practice.

However, larger scale of studies are needed to build a robust model, and a lot of future work is needed to optimize the flow cytometry risk scores for clinical application. Single-cell RNA sequencing (scRNA-seq), which comes with high cost hindering its application for large-scale prognostic analysis, nonetheless has revealed subclonal heterogeneity, including the drug-sensitive and -resistant DLBCL cell populations separated by CD48 and CD62 markers, as well as heterogeneity of T-cell populations (e.g., cytotoxic, Th1, Th2, regulatory, PD-1+ T cells) (13) which may explain why in the flow cytometry study by Yu et al, after excluding patients with low T cell percentages, high T-cell percentages did not show further prognostic significance. Normal B cells are also heterogeneous, and a scRNA-seq study in non-small cell lung cancer found that tumor-infiltrating naïve-like but not plasma-like B cells were predictive (14). Hence, it is necessary to delve into the heterogeneity of each cell type and the crosstalk between lymphoma and immune cells, and to further establish practical flow cytometry marker panels and gating strategies for isolating tumor/immune cell subclones. Moreover, as new techniques such as phosphoflow cytometry have been applied in clinical studies (15–17), flow cytometry-based prediction models can be substantially improved in the future. Markers of tumor and TME interaction can also be integrated into the standard prognostic scoring systems as proposed in chronic lymphocytic leukemia (18).

The limitation of flow cytometry quantification should also be recognized. In the study by Yu et al, only cases with abnormal B cells successfully identified by flow cytometry were selected, thus excluding DLBCL cases missed by flow cytometry, which occurred in 3-30% of DLBCL cases (19–25). The median percentage of overall B cells in the study cohort was still lower (also lower macrophage percentage while higher T-cell percentage) than that by fluorescent multiplex immunohistochemistry. The authors also suggested that gating strategies and light chain kappa/lambda restriction analysis may not always accurately isolate normal B cell populations (6). Similarly, scRNA-seq results may not truly represent macrophage subpopulations (26). Another disadvantage of flow cytometry data is the incapability to identify the ‘mesenchymal’ category of lymphoma TME, which features abundant lymphatic and vascular endothelial cells, fibroblasts, fibroblastic reticular cells, and extracellular matrix (10).

## Increased normal B cells as a novel favorable prognostic factor in DLBCL

Compared with tumor-infiltrating T cells, tumor-infiltrating B lymphocytes (TIL-Bs) are less studied. In recent years, multiple studies have shown favorable prognostic effects of TIL-Bs in solid tumors (27, 28). Flow cytometry can distinguish normal B cells from lymphoma B cells, and Yu et al. (6) have validated the favorable prognostic association of TIL-B abundance determined by flow cytometry in DLBCL patients, which was first shown in an International Lymphoma Consortium cohort through ultra-deep sequencing of immunoglobulin genes (9). In that study by Xu-Monette et al, higher frequencies of normal B cells in total B cells determined by genetic assays were associated with significant prognostic effects in overall DLBCL, DLBCL cell-of-origin subtypes, and the EZB genetic subtype. These novel findings may provide a basis for developing more effective immunotherapies. For example, cytokines promoting interaction between normal B cells and T cells may enhance the efficacy of chimeric antigen receptor T cell therapies. If antibodies secreted by TIL-Bs can be identified, they could be used to target tumors while spare normal B cells, thereby having advantage over rituximab, anti-CD19 antibodies, antibody-drug conjugates, and engineered chimeric antigen receptors currently used in DLBCL treatment.

Further mechanistic and therapeutic studies using other approaches are warranted. First, we need determine whether B-cell functions in cell-mediated or humoral immunity, or both, are responsible for the observed superior survival. To be more specific, whether antigen-presentation function of B cells, contribution to optimal activation of T cells, cytokine production, or antibody-dependent mechanisms are more critical (27). Second, we should examine B cell functions and subsets (e.g., naïve, germinal-center, and memory B cells, antibody-secreting plasma cells), as well as the differences between DLBCL subtypes. Third, we need determine whether and how therapies influence the quantity and antitumor function of TIL-Bs. In the study by Yu et al, TIL-Bs showed more remarkable prognostic effects in treated patients, especially those receiving upfront rituximab.

## Discussion

In summary, Yu et al. showed that in a single-center DLBCL cohort, real-world flow cytometry data of tumor-infiltrating T, NK, and B cells had predictive values, which has important clinical and therapeutic implications. We look forward to future clinical and mechanistic investigations of TIL-B functions, both of which hold paramount importance for developing a robust clinical decision support tool and novel therapies for DLBCL.

## Author contributions

ZS: Conceptualization, Writing – original draft. RT: Visualization, Writing – review & editing. HW: Writing – review & editing, Methodology. XF: Conceptualization, Funding acquisition, Writing – original draft.
